# Factors Influencing Attendees’ Engagement with Group Psychoeducation: A Multi-stakeholder Perspective

**DOI:** 10.1007/s10488-021-01182-y

**Published:** 2022-01-06

**Authors:** Agnes Higgins, Carmel Downes, Rebecca Murphy, Jennifer Barry, Mark Monahan, Louise Doyle, Patrick Gibbons

**Affiliations:** 1 School of Nursing & Midwifery, Trinity College Dublin, 24 D’Olier Street, Dublin, Ireland; 2 Department of Psychology, National University of Ireland, Maynooth, Kildare, Ireland; 3 Celbridge Adult Mental Health Services, Kildare, Ireland

**Keywords:** Psychoeducation, Engagement, Service users, Families, Enablers, Barriers, Qualitative design

## Abstract

Few studies have explored the problem of engagement in relation to group psychoeducation from a multi-site and multi-stakeholder perspective. The aim of the study was to explore the factors influencing service user and family engagement with group psychoeducation programmes. The study design was qualitative descriptive. Data were collected through individual and focus group interviews with key stakeholders (n = 75) involved with the programme within 14 mental health sites in the Republic of Ireland. Enablers and barriers to engagement were identified at participant, provider, programme and organization level. Motivated participants and engaged clinicians, peer co-facilitation and support, and skilled and responsive facilitators were some of the factors which enhanced engagement. Barriers to engagement included readiness among participants, concerns related to stigma and confidentiality, desire to distance oneself from mental health services, a lack of support for programme participation within families, group discomfort, the time and length of the programme, issues with transport, visibility of the programme, and structural supports for clinicians. Findings from the study illustrate the multifaceted nature of engagement as well as provide a greater understanding of the multifactorial influences on engagement. Strategies to enhance engagement should therefore reflect a multipronged approach. At the outset of programme implementation, organizations should address their readiness to engage, conduct local needs assessments to anticipate individuals’ needs and plan accordingly in order to maximize engagement, and bolster facilitators’ engagement skills through the provision of training and mentoring opportunities.

**Supplementary Information:**

The online version contains supplementary material available at 10.1007/s10488-021-01182-y.

## Background

The distress associated with mental health problems such as schizophrenia or bipolar disorder is well documented from the perspective of the person experiencing it (Crowe et al., 2012; Warwick et al., 2019) as well as from family members’ perspectives (Cleary et al., 2020; Young et al., 2019). A central pillar underpinning evidence-based care for people with such a diagnosis is the provision of psychoeducation. Psychoeducation as an evidence-based intervention is included in international and national clinical guidelines on the care of people experiencing psychosis (Institute and for Health and Care Excellence (NICE), 2014; Health Service Executive (HSE), 2019). Numerous randomised controlled trials and systematic reviews attest to the clinical and recovery benefits of service users engaging with psychoeducation, including decreased relapse rates, increased awareness of symptom triggers and warning signs of distress, strengthening of self-efficacy, hope and sense of empowerment as well as greater utilisation of mental health services (Druss et al., 2010; D'Souza et al., 2010; Pickett et al., 2010).

A high percentage of people experiencing psychosis continue to live with or are in regular contact with family members, who not only provide emotional and practical support but play a significant role in accessing services when someone becomes unwell (Hackethal et al., 2013). In light of this the provision of education to family members is also considered best practice (Health Service Executive (HSE), 2019; Institute and for Health and Care Excellence (NICE), 2014). Positive engagement of family members with psychoeducation programmes not only enhances their knowledge of mental health problems and the supports available, but has been linked with interrupting negative patterns of interaction between the person and family members (Jewell et al., 2009; Miklowitz & Chung, 2016; Rummel-Kluge & Kissling, 2008; Sin et al., 2013; Taylor et al., 2009). In addition, psychoeducation has been shown to positively impact carer burden, coping capacity and problem solving in crisis situations (Brady et al., 2017). Consequently, supporting engagement of service users and family members needs to be a central pillar of all psychoeducation programmes.

A lack of engagement in treatment among those with mental health difficulties is a significant problem (Crocker et al., 2020). Previous research indicates that at an individual level, factors such as male sex, younger age, greater severity of illness, diminished functional capacity, having a diagnosis of schizophrenia or schizoaffective disorder (Dixon et al., 2016; Doyle et al., 2014; Kingston et al., 2018; Kreyenbuhl et al., 2009; Lynch et al., 2019; Medalia & Saperstein, 2011; Sin Fai Lam et al., 2016) and comorbid mental health and substance use conditions (Dixon et al., 2016; Lynch et al., 2019) are associated with higher rates of attrition and disengagement from mental health services. Perceptions about the efficacy of treatment (Lynch et al., 2019) as well as the quality of the interactions between clients and providers (Lynch et al., 2019; Tindall et al., 2019) can also inform decisions about whether or not to engage.

Engagement with a psychoeducation programme is not just about enrolling, recording attendance and attrition, but rather is a dynamic, co-constructed process (Bright et al., 2015) that moves along a continuum from recruitment, active participation, to sustained engagement for duration of the programme. In line with this thinking, engagement focuses on buy-in and emotional investment from service users and families (Kim et al., 2012) as well as the facilitators’ role in building collaborative relationships (Lizardi & Stanley, 2010; Tindall et al., 2018) and supporting engagement through their facilitation style (Bright et al., 2015). The National Alliance of Mental Illness (Lynch et al., 2019, p. 79) not only emphasises the importance of individuals’ forging alliances with providers but draws attention to the importance of considering engagement within the context of family and wider supports.

While a significant body of work exists on the benefits of delivering psychoeducation, few studies specifically explore factors that influence engagement with group psychoeducation post-intervention. The studies that have reported on aspects of engagement suggest that participant, provider and intervention factors have an influence. These include participant factors, such as mood of service users (Poole et al., 2015), competing demands (Petrakis et al., 2014; Poole et al., 2015), difficulties in group situations, and concerns for confidentiality (Hackethal et al., 2013). Provider-related factors include clinicians’ belief in the value of group psychoeducation (Ingvarsdotter et al., 2016) and skills of facilitators in supporting participant engagement (Coulthard et al., 2013; Poole et al., 2015). While these studies provide useful insights, they are limited by small sample sizes (7–18 participants) and in many cases exploring factors that influence engagement was not the primary aim of the study. In addition, the studies collected data from one site and one stakeholder group, such as facilitators/programme leaders (Coulthard et al., 2013; Ingvarsdotter et al., 2016; Whitley et al., 2009), clinical staff and agency directors (Whitley et al., 2009), service users (Poole et al., 2015) or family members (Petrakis et al., 2014), thus potential differences or conflicting perspectives between participants and facilitators were not examined. Given the benefits of psychoeducation, and the potential for poorer health outcomes and increased healthcare costs associated with disengagement (Crocker et al., 2020; Dixon et al., 2016), understanding factors that influence attendee engagement is critical.

This paper aims to address this research-to-practice gap by generating evidence on factors that influence attendees’ (participants’) engagement from a multi-site and multi-stakeholder perspective in order to identify and develop strategies to increase engagement. The focus of the paper is on the perspectives of those either participating or directly involved in delivering a psychoeducation programme rather than the views of higher-level policymakers. The data which this paper is based on is part of a larger funded study into the structural, cultural and systemic processes that have enabled or hindered the adoption, implementation and sustainability of group psychoeducation programmes within the mental health system (Higgins et al., 2020).

## Methodology

### Aim

The aim of this current aspect of the study was to explore the factors influencing service user and family engagement with group psychoeducation programmes, called respectively the EOLAS Programme for Service Users and the EOLAS Programme for Families and Friends (EOLAS is the Irish word for knowledge).

### EOLAS Programme

The content, format and delivery of the EOLAS programmes are co-designed, co-developed and co-facilitated by service users, family members, and clinicians (Higgins et al., 2017a). The EOLAS Programmes were first piloted in one mental health service in Kildare in 2011 and, following positive evaluations (Higgins et al., 2017b, 2019, 2020), were extended to 14 further mental health services around Ireland. The group programmes consist of eight weekly sessions of approximately 90-min duration with some sessions being delivered by a guest speaker which is decided in collaboration with the participants. In addition to facilitators having a manual to guide them through the programme, all facilitators (clinicians and peers) undergo a four-day training programme, to support the development of co-facilitation skills and inform them about the programme. Potential participants are referred to the programme by members of the multidisciplinary Community Mental Health Team and once they commence the programme they also receive a manual, which they can use as a resource during and after completing the programme. A more complete description is available in the following paper (Higgins et al., 2017a) and within the programme website (https://eolasproject.ie/).

### Methods

The research design that informed this aspect of the study is qualitative descriptive. As rich, detailed, and context specific data were needed to answer the research aim a qualitative descriptive approach was considered appropriate. Emphasis within qualitative descriptive research is on staying close to the “surface of the data and events” and on providing a rich description of the phenomena in easily understood language as opposed to a highly abstract rendering of data required with other qualitative designs (Bradshaw et al., 2017, p. 2). The research is reported in accordance with the consolidated criteria for reporting qualitative research (COREQ) checklist (Tong et al., 2007).

### Data Collection

Data were collected through semi-structured interviews, guided by an interview schedule and audio recorded with permission. Potential participants were given the option of participating in either an individual or focus group interview. In light of the limited resources available and a wish to capture the views of as many participants as possible from services that were geographically some distance apart, it was decided to collect data using both a focus group interview and an individual interview. The focus group also enabled the collection of data from potential participants who were meeting as a group for other EOLAS related issues. The focus groups were grouped according to role in the EOLAS programme (Co-ordinator, facilitator, attendee).

The team developed an interview schedule to guide data collection on participants’ views of the factors they believed enabled or hindered the implementation of the intervention and part of the schedule included the topic of barriers and enablers to attendees’ engagement (Additional File 1). To ensure consistency in data collection the same schedule was used for both the individual and focus group interviews and reviewed by some of the research team after the first round of interviews. Two members of the research team (RM and JB), who were not well known to the participants, collected the data between late 2018 and 2019. Both interviewers were female; one was a postdoctoral researcher with extensive experience in qualitative research and the other had an academic education in psychology.

### Recruitment

Based on their ability to inform the study objectives, a purposeful sample of participants were selected from 14 mental health services involved in delivering the intervention. Emphasis within recruitment was on interviewing groups ‘deemed rich in information for the purpose of saturating the data’ (Lambert & Lambert, 2012, p. 255). Potential participants (co-ordinators, facilitators, programme attendees) who had previously provided consent to be contacted by the project team were sent an information brochure, together with a letter requesting them to contact a member of the research team if they were willing to participate. The information brochure contained the aims of the research and information on data collection and the consent process. Once a potential participant made contact, all questions were answered and a time for either an individual or focus group interview was arranged.

Focus group interviews were conducted face to face, whereas the individual interviews, depending on participants’ preferences, were either face-to-face or by phone. The focus group and individual face to face interviews took place in a hotel, mental health service or university. In total, 8 focus group interviews and 42 face to face individual interview were conducted. The duration varied between approximately 30 min to 1 h, with all the focus group interviews being approximately one and a half hours duration. Fieldnotes were recorded after each interview. Participants were informed that they could review the transcripts if they so wished, but no participant took up the offer.

### Ethical Approval and Access to Participants

Ethical approval for the study was granted by the University’s Research Ethics Committee and the ethics committees of the services involved. All participants received written and verbal information about the study and provided written consent prior to the interviews.

### Profile of Participants

75 people participated in the study, 42 in one-to-one interviews and 33 in focus group interviews. Participants included EOLAS co-ordinators (n = 16), EOLAS facilitators (clinical n = 12; peer n = 25), programme participants/attendees (n = 16) and other key stakeholders (n = 6). The other stakeholders included senior managers of the Health Service Executive, members of the EOLAS steering group and project workers who had responsibility for coordinating the national roll out of the programme, under the direction of the steering group. More detailed information on the profile of interviewees is given in Table 1.

**Table 1:** Profile overview of interviewees by role in EOLAS and method of data collection

Role in EOLAS	Individual interview (n)	Focus group (n)	Demographics and background information
EOLAS clinical facilitators	2	10	*Gender:* F = 10; M = 2 *Profession:* Nurse = 6; Social Worker = 4; Occupational Therapist = 2 *Years working in MHS:* Mean = 15.58, SD = 8.93, Range = 4–29 years *Years involved with EOLAS:* Mean = 4.5, SD = 2.38, Range = 1–6 years *Number of EOLAS programmes delivered:* Mean = 3, SD = 1.83, Range = 1–5
EOLAS coordinators* *12 had experience of facilitating the EOLAS programmes	7	9	*Gender:* F = 11; M = 5 *Profession:* Nurse = 8; Social Worker = 7; Psychiatrist = 1 *Years working in MHS:* Mean = 16, SD = 11, Range = 3–40 years Years involved with EOLAS: Mean = 3.43, SD = 1.89, Range = 1–7 years
EOLAS peer facilitators	11	14	*Gender:* F = 15; M = 8 *Background:* Family Member = 11; Service User = 14 *Number of EOLAS programmes delivered:* Mean = 2.29, Range = 0–7 *Number of facilitators currently facilitating EOLAS:* n = 18
EOLAS participants/attendees	16		*Gender:* F = 11; M = 5 *Background:* Family Member = 12; Service User = 4 *Length of time since EOLAS completion:* < 1 year = 8, > 1 year = 7
Other key stakeholders	6		*Gender:* F = 2; M = 4 *Background:* EOLAS Steering Group members = 3; Project Workers (former and current) = 3

### Data Analysis

**Fig. 1:**
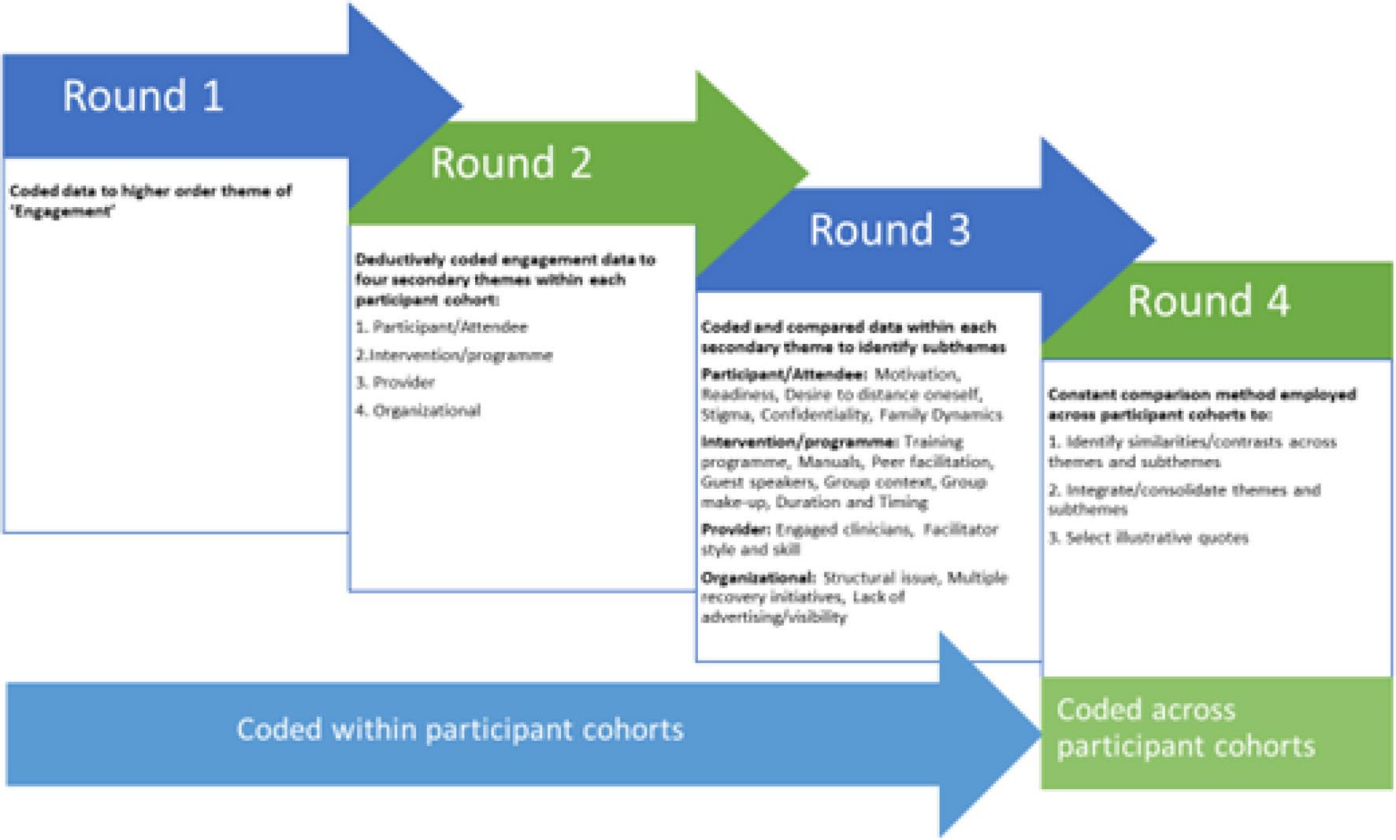
Phases of the analytical process

Interviews were transcribed and uploaded to NVivo12 for analysis (QSR, 2018) Prior to analysis all transcripts were read and reread, so that the team involved in the analysis were familiar with the transcripts. The analytical process moved through several phases (Fig. 1), using both a deductive and inductive approach.

The first round of analysis involved coding any data in each interview under a higher order theme called engagement, and this was completed separately for each group of participants. Engagement was defined on a continuum ranging from initial information giving about EOLAS as an aspect of recruitment, through to sustained active participation with the programme.

Once the first round of analysis was completed for all groups, a second round of coding was completed, again at group level. This involving using template analysis and coding each groups’ data using the following four secondary themes: participant/attendee, intervention/programme, provider and organizational. Subsequent to this, phase three commenced. Data coded to each secondary theme was explored in greater detail, and using an inductive process the factors impacting engagement (subthemes) were identified and named.

Once this process was competed for each group, the subthemes from all groups were merges in order to compare and make explicit similarities and differences in views across participant groups. The final aspect of this phase of analysis involved reviewing the final structure and selecting illustrative quotes (Additional file 2).

### Trustworthiness

Trustworthiness, the qualitative version of rigour addresses issues credibility, dependability, confirmability, transferability, and reflexibility. Table 2 provides a summary of the provisions made by the researchers in this study to address these issues and to ensure credibility, transferability, dependability and confirmability of qualitative data.

**Table 2:** Trustworthiness of data

Quality criterion	Provision made by research team
Credibility	Inclusion of different stakeholder groups (service users, family members and clinicians) from variety of mental health services and geographic location Triangulation of data across different groups Use of quotes to support description of phenomenon (barriers and enablers to engagement)
Dependability	In-depth description of study methods to enable replication Use of a coding framework
Confirmability	Analysis completed by more than one person. CD and RM independently coded all data in round 1 and 2. Round 3 and 4 were completed as a team process to agree consensus around interpretation (RM, CD and AH) Codes and themes discussed between researchers to minimise interpretative bias and achieve consensus
Transferability	Description of research setting, participant profiles provided
Reflexivity	Care was taken to work reflexively, question interpretations and assumptions and minimise interpretative bias Questioning about biases was helped by the fact that the research team came from different disciplinary backgrounds, including sociology, social science research, psychology, psychiatry, and mental health nursing

## Results

Participants described a range of factors that impacted engagement within each domain. Some of these were specific to a particular phase of the engagement continuum, while others impacted on more than one phase, and some were both an enabler and barrier. Although some factors generated a greater number of coded units (pieces of data) in comparison to others, in the paper we include all identified as it is the totality of factors and views that need to be considered in designing interventions to enhance engagement.

**Fig. 2:**
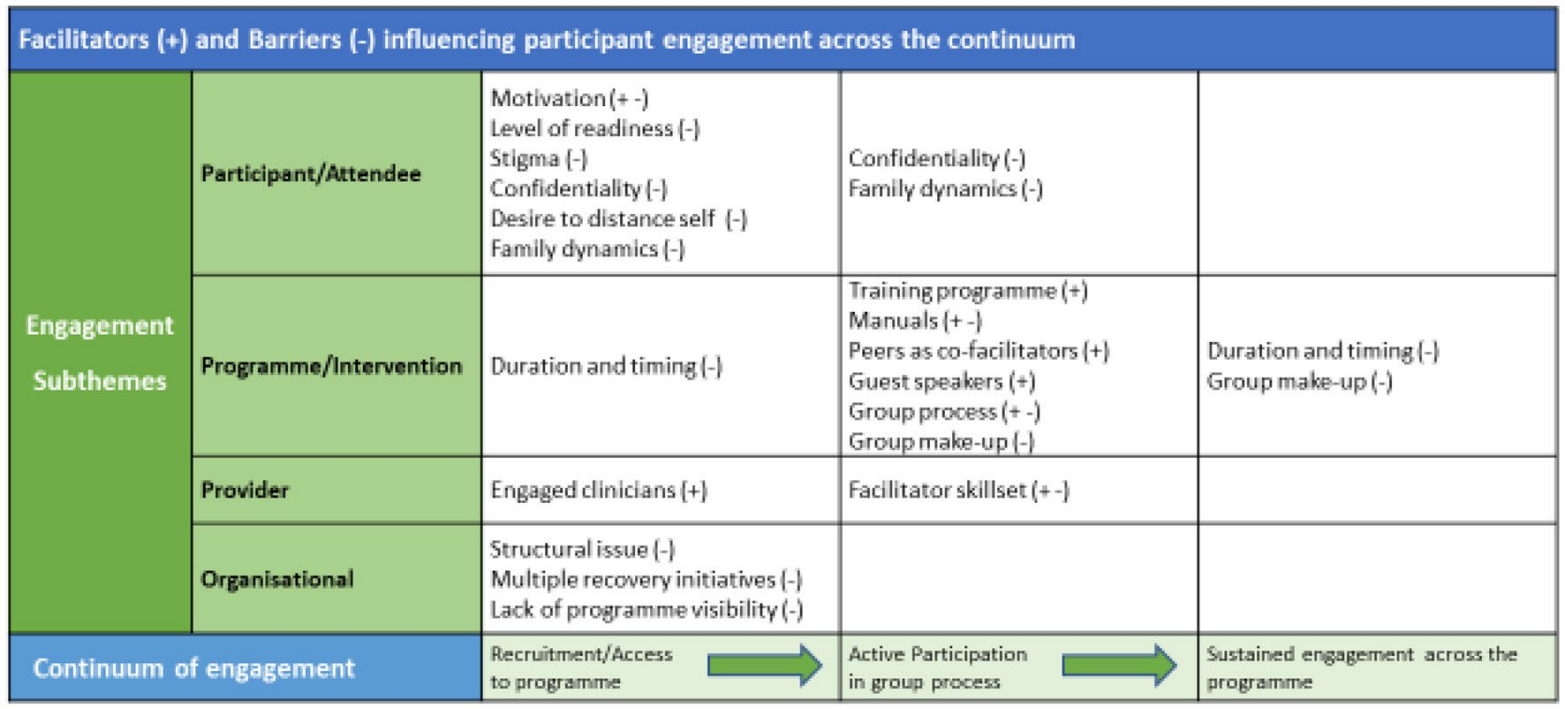
Factors influencing participant engagement across continuum

Figure 2 provides an overview of factors that impact across the continuum and indicates the nature of the impact.

### Participant/Attendee Related Factors

A number of participants/attendees related factors were identified as impacting uptake and engagement with EOLAS, namely, motivation, level of readiness, desire to distance oneself, stigma, confidentiality, and family dynamics.

Overall, being motivated by the desire to learn more about mental health, coping strategies and how the mental health services could support and help service users and family members to move forward in their recovery or support a family member in recovery was viewed as a key enabler to engagement. In contrast, the lack of motivation, which sometimes reflected a broader pattern of disengagement from services, or a lack of desire to change current circumstances or situation was viewed as a barrier. In particular, clinical participants suggested that service users who were involved with the mental health services for a number of years may not see the programmes as offering anything new in terms of learning.

The service users in one setting or another had heard a lot of the information before. And the buy in from them was a little bit more difficult. They wouldn’t always come, they’d be but I’ve heard this a hundred times. You know they weren’t as interested. CF11 (FG)

In contrast family and service user participants suggested that a key barrier in terms of motivation was the failure of services to sustain new initiatives, which resulted in them (family and service user attendees) not wanting to invest time in another intervention that might not be sustained within the mental health care system.

I know service users get very disheartened. Because they say, oh that’s another group, a fly by night. And that’s why people don’t get involved. Because so many things have come to the wall over the years. FF11 (FG)

The second factor was readiness of attendees. Clinical participants in particular noted that service users who were at the beginning stages of their recovery journey, particularly those who were newly diagnosed or recently discharged from hospital, needed to be ‘well enough’ to benefit from the programme. They also noted that families too needed a period of time to adjust to and accept their relative’s situation before coming on the programme.

I mean if you have someone who is just post discharge or you know actively unwell, you know something like this for some people mightn’t be very helpful. CF1 (II)

The family members, it’s often years down the line when they’re really ready for something like that [EOLAS]. CO2 (II)

While family and service user participant didn’t disagree with the importance of readiness, service users and service user facilitators hypothesised that it was not a lack of readiness but more a desire to distance oneself from the mental health services once discharged from inpatient care.

Because when you get better and when you get out of hospital, you’re kind of thinking I don’t want to associate with that [hospital]. SF4 (II)

Other barriers identified included attendee fears in relation to the stigma associated with mental illness, with some participants recounting how they or other participants because of the stigma opted to attend programmes outside their immediate locality.

[...] we were running two groups. One in [rural town] and one in [Urban town]. And a couple of people decided that they would travel the twenty miles to other group, you know, those are just sensitivities that are there. CF5 (FG)

Another factor identified was fears and concerns related to confidentiality of the information shared during the programme. It was noted that disclosing personal details and experiences was an exposing and vulnerable experience for some participants, particularly family members.

[...] there was a lot of anxiety about the family members coming into a room with other family members, they almost felt that they were exposed […], felt very vulnerable coming into that space, safety [about information]and all that. So there was a barrier […] the service users were more accustomed to being among one another, in waiting rooms and clinics, so had less of that concern about being exposed. CF7 (FG)

Family dynamics were also viewed as impacted engagement. Participants reported that in some cases, family members experienced disapproval or anticipated disapproval from their relative with mental health issues, and this disapproval prevented or made them reticent to participate in the family programme.

[…] a lot of families saying, oh I don’t know about that and whether the service user, family member would like it. CF2 (II)

### Programme/Intervention Related Factors

The domain focuses on aspects of the EOLAS intervention. Several aspects of the programmes were identified as influencing participants’ active participation and ongoing attendance and engagement, such as the training programme provided to facilitators, programme manuals, peer facilitation, guest speakers, group context, group make-up, and duration and timing. While some were enablers to engagement, other aspects were perceived as barriers to depth and quality of participation.

Many of the facilitators expressed the view that the training, and in particular the exercises and role play, which were part of the training not only enhanced their understanding of the programme content and facilitator role but imbued them with confidence and increased their readiness to engage actively with participants during the delivery of the programme.

The huge strength of it was train the trainer training… we covered the course content and were being trained as to how to deliver those modules. FP6 (II)

All groups, but in particular service user and family member participants regarded the programme manuals as being a useful source of information on a range of topics. Having the manuals was important not just as a guide to the programme, but served as a useful reference point for information after the programme ended. Some family members used the manual to facilitate communication within the family after the programme ended.

You would go home to look back on it [manual]. I would leave the book open and say to my son did you see that page. Did you look at that you know so it triggers us to talk about different things. FP11 (II)

Manuals were viewed as an enabler to engagement when they were used within the group to stimulate and facilitate valuable insights to emerge from the attendees. In contrast, an over-reliance on the programme manuals was viewed as a barrier to engagement by closing down vital conversation within the group.

But it [the manuals] can feel somewhat prescriptive at times when the conversation might lead elsewhere, so need to be flexible. CF7 (FG)

All stakeholder groups viewed the involvement of peers in the co-facilitation as a positive aspect of the design which enhanced engagement and participation. The peer element was seen as central to breaking down barriers and equalising power relationships. For some this may be the first time that they had the opportunity to talk to someone with the same diagnosis or circumstance as themselves. In addition, the peer facilitators, created a friendly, reassuring atmosphere, inspired hope and spoke a language that participants could understand and identify with.

Well I found that [co-facilitation] helpful because there was a person delivering the course that had mental health issues. So they would have insight to what it’s like to have mental health difficulties and their understanding would be better, they’d be talking and able to relate their own experiences as well. SP1 (II)

The guest speaker sessions, particularly those involving psychiatrists and pharmacists, were also viewed as enhancing participants’ ongoing attendance. Being provided with an opportunity to get a concentrated period of time with these practitioners, pose questions and receive answers was really valued by both service user and family participants.

It brought the, the top people down to our level and we were able to relate to them and it didn’t feel like they were up at the top and you couldn’t reach them […] we were encouraged to tell them how we felt, and ask about different medications. FP7 (II).

The discursive part of the group sessions was considered both an enabler and a barrier. The opportunity to share experiences and learn from each other in a supportive environment was viewed as vital to engagement and provided an important mechanism for peer support. However, for some, opening up within a group context was a challenge, particularly family members who were unaccustomed to disclosing in a group context. It was also noted that this lack of participation by some attendees made it difficult to engage the group as a whole and possibly deterred other attendees from opening up.

[…] even though all the family members did benefit a lot. The hardest thing was getting [them] to talk … they were very reluctant to come forward and kind of talk. FF10 (FG)

Participants also perceived that the representativeness in some groups’ demographic make-up negatively impacted engagement. Most family groups comprised parents, with spouse or sibling participants being in the minority, which inhibited engagement. Others commented on how gender imbalance and age differences within service user groups may also have impeded engagement and contributed to attrition.

I suppose one of the things that may have had an impact is, we had 2 females and 8 males and then one female couldn’t really attend due to work commitments. So we were left with one lady with a lot of males. So I feel that might have impacted her attendance going forward. CF1 (II)

The final programme related factor was the duration of the programme and timing which were perceived as a barrier for some potential attendees. Some people could not commit to the eight-week programme due to obligations related to family, work and education or were experiencing challenges due to transport, particularly in rural areas.

I think there’s a better chance of getting people for shorter periods. […] just even at weekends […] if we had the 3 options over the 8-week period, over 4 weekends whatever way we break it down […] that’s what we’re thinking of going forward. CO4 (II)

### Provider Related Factors

The provider domain focuses on aspects of the individual providers who were involved in recruitment and subsequent facilitation of the programme. Within this domain two key factors were identified: engaged clinicians and facilitators’ skillset. There was general agreement that a key enabler to recruitment was the involvement of motivated local clinicians who had well established relationships with, and knowledge of, service users and families. Having prior knowledge and relationships meant they were able to promote EOLAS and encourage engagement based on the needs and context of people lives. Being willing to promote the programme and follow up potential attendees on an ongoing basis, as opposed to simply giving once off information, was viewed as an essential. An absence of engaged clinicians was seen as adversely affecting potential participants’ access to and engagement with EOLAS.

The key worker to go out to the service user or the family member and say come on, this is great, I’ll come with you, I’ll be there on the first night and all, and then they will, they might buy in because they’ve had a long standing relationship maybe with that person and they trust that they have good judgment in this being valuable to them, and that was really the key to kind of getting people to come along and trust that it was going to be a good process. CO13 (FG)

Once the programmes commenced, facilitation skillset and style were identified as being instrumental to engaging participants. Participants identified that good communication skills and an openness to sharing personal experiences were required to cultivate rapport and successfully engage attendees within the groups. Facilitators and attendees also spoke of the importance of facilitators having the ability to adopt a flexible approach to facilitation in order to engage responsively with participants’ informational and emotional needs, as well as having the ability to use strategies such as breaking out into smaller groups to engage people who were reticent about contributing to group discussion.

But you need knowledge of how to facilitate… how to do that well, and co facilitate and an expertise to be able to understand that the dynamics and how to work with it and all that sort of stuff. […] your experience of the product [programme] is going to be as good as the facilitation… what happens in the room. SH1 (II)

In addition to have communication skills and being able to uses various facilitation strategies, facilitators highlighted the importance of the clinical and peer facilitators knowing each other and being comfortable working in harmony to promote a positive group atmosphere and not allowing difference of opinion or perspective to become a focal point of tension was seen as important enabler of engagement.

I got on well with both the clinicians that were there. Because I would’ve done a lot of work with them kind of before EOLAS. So we were pretty comfortable working with each other. And I suppose it kind of; it probably makes the rest of the group that are there feel more relaxed. Because we kind of were able to bounce off each other. And it seemed quite smooth. So I think definitely having to get to know the other person you’d be working with is kind of quite important. Just so you can, you get to know each other’s style. SF6 (II)

### Organizational Level

Within the organisational domain the following were all identified as barriers: structural issues, multiple recovery initiatives, and lack of advertising/visibility of EOLAS programmes. The clinical participants in particular identified several structural barriers to promoting attendees engagement. Workload, lack of protected time, and involvement with other initiatives impacted some clinicians’ capacity to engage with the recruitment process. In addition, clinicians noted that where there are a multitude of recovery initiatives in addition to EOLAS, it was difficult for all clinicians to keep EOLAS on the agenda as some services and clinicians prioritised and invested their time in other initiatives.

We have 2 people, clinicians who are trained [in EOLAS] and who will run it but they are not championing it because they are championing other things and everybody cannot champion everything. CO5 (II)

Clinicians also reported that the competition between recovery initiatives diverted potential participants away from EOLAS and depleted the recruitment pool,

I know there in this region there’s the early intervention for psychosis and that’s kind of taken over, which is putting EOLAS in a difficult kind of position. SF4 (II)

In contrast family member and service user facilitators were of the view that it was a lack of advertising, awareness and visibility around EOLAS, rather than a lack of participants or need, that was a barrier to participant engagement.

So I think it’s a visibility within services issues. And I think it’s up to the services to work on increasing that visibility. SF1 (II)

## Discussion

Even under optimal internal organisational conditions, a key challenge facing mental health practitioners is connecting and engaging with service users who experience psychosis and their family members. The findings from this study highlight how the challenges of connecting and engaging with service users/family members is not simply located within participants’ attitudes, personal preferences, and the wider context of their lives, but also encompasses a wider issue of how facilitators’ perspectives and skills, the internal characteristics of the programme, model of delivery and organizational context can enhance or constrain engagement. While there was convergence of opinions in some areas, the findings also reveal differences of perspectives and emphasis, such as clinical participants emphasis on attendees’ readiness, motivation and willingness to engage, whereas service users and family members emphasized lack of visibility of the programme, failure of services to sustain new initiatives and a desire by service uses to distance themselves from mental health services once discharged.

It is noted in other literature that clinicians as ‘gatekeepers’, sometimes limit access of service users and family members to evidence-based interventions (Deane et al., 2006). Murray-Swank et al. (2007) noted how a lack of invitation from clinicians was a barrier to family participation, with Ingvarsdotter et al. (2016) commenting on how some clinicians were ‘selective’ in their recruitment i.e. only informing people they thought would attend and avoiding people they felt would be uncomfortable within a group. While these issues did not emerge as a major issue in the delivery of the EOLAS programmes, organisational issues such as the multiple recovery programmes being simultaneously run within services, heavy workloads and a lack of protected time adversely impacted clinicians’ capacity to dedicate time to promote and advertise the programme and engage with potential participants. These barriers require greater organisational leadership in the effective management of resources in order to enhance clinicians’ capacity to promote the intervention and increase its visibility to potential participants as well as to members of the multidisciplinary team. Greater visibility of the programmes among potential participants may result in them seeking out the programme rather than waiting for clinicians to initiate engagement.

The findings also highlight how successful engagement of participants in psychoeducation interventions is not a once off event but required time to build the relationship between clinicians and service users/family members, as well as a willingness by clinicians to engage continually and to follow-up with participants. The importance of establishing relationships with potential participants in order to improve service users’ and families’ understanding of interventions and to address any concerns is also highlighted elsewhere (Ingvarsdotter et al., 2016; Lynch et al., 2019). Outreach work with service users and families is important not only to build rapport, provide information and address any barriers, but it enables clinicians to assess individuals’ readiness and ability to engage in the intervention. Outreach encounters also offer valuable opportunities to address differing perspectives on factors which inhibit engagement and to explore ambivalence and the pros and cons of attendance. Additionally, clinicians can use this opportunity to identify, explore and resolve family dynamics which this study found inhibited some individuals from engaging in the intervention. Sherman and Fischer (2012) also found that service users were opposed to their family taking part in a group education intervention for fear that their personal issues would be discussed outside of the group causing them embarrassment, while service users in Lucksted et al.’s (2012) study expressed discomfort with the thought of family members talking about them in their absence. This opposition highlights the importance of clinicians emphasising to service users the value of family education in supporting families to learn skills to cope, problem solve and understand the experience of severe mental health illness (Duckworth & Halpern, 2014; Sherman & Fischer, 2012).

Issues around stigma and confidentiality were also highlighted as being barriers to engagement. To assuage these fears and create a safe environment, assurances around confidentially should be explicitly and repeatedly outlined during recruitment and as an ongoing part of the programme. In addition, the findings highlighted that some individuals, particularly family members, have difficulties being emotionally vulnerable and disclosing personal feelings in a group format. Similar difficulties have been reported in other studies. Family members and careers in Petrakis et al.’s (2014) study reported that discomfort in social situations and revealing emotions and personal experiences, and having competing commitments all acted as barriers to participation in a group intervention, with clinicians in this study identifying fear of the unknown, difficulties communicating emotions and logistical challenges around time and location as inhibiting engagement. Similarly, other studies reported that feelings of stigma, embarrassment and discomfort, as well as the presence of domineering group members, were challenges to engagement in group psychoeducation interventions (Lucksted et al., 2012; Poole et al., 2015; Sherman & Fischer, 2012).

While clinical and peer facilitators in our study identified the importance of small group work and informal group chat as potential strategies for engaging more reticent participants, it must be recognised that group psychoeducation interventions may not be suitable for all individuals. Therefore, alternative formats, such as individual one-to-one psychoeducation, should be available to accommodate individuals who are not ready to engage in group work in order to ensure that they receive the support they require.

Like several other studies (Hackethal et al., 2013; Petrakis et al., 2014; Poole et al., 2015; Sherman & Fischer, 2012), our study found that the timing of the programme in relation to work and other commitments, as well as access to transport, impacted whether individuals could engage. To address these issues, a needs assessment should be conducted in order to ascertain individuals’ preferences regarding the timing and duration of an intervention as well as any needs they have in relation to transport. In addition, study findings point to the need to explore alternative routes to inclusion, such as self-referral to a co-ordinator who could then inform the potential participant about the programme, as well as the need for organisations to conduct local needs assessments routinely in order to ascertain the need for psychoeducational interventions given the target cohort for EOLAS in some areas was not sufficient.

While greater flexibility in delivery might make it more convenient for service users and families to engage, the challenge for manualised psychoeducation programmes such as EOLAS is to identify the ways in which it can be adapted while at the same time ensuring that the fidelity and efficacy of the intervention is preserved. Irrespective any change should follow a systematic process, informed by implementation science literature (Kirk et al., 2020; Stirman et al., 2019). One possible adaptation that has demonstrated utility in terms of overcoming some of the personal and practical barriers encountered to service user/family engagement is telehealth (Dixon et al., 2016) and this should be considered for the EOLAS programmes, especially given the challenges currently being experienced in delivering group learning experiences while respecting the limitations of social distancing imposed by the ongoing COVID pandemic.

Bearing in mind that any adaptation to a programme is a finely balanced task requiring trade-offs and compromises, this study shows that there are some aspects of group psychoeducation which are integral to participant engagement and should be promoted and preserved; namely peer co-facilitation, the opportunity to share experiences with and to learn from the group, as well as the opportunity to engage with mental health professionals, but with valued clinical input being delivered in conjunction with lived experience in a way that respects the value of both sources of expertise.

Similar to other studies, peer involvement which is central to all mental health policy (Institute and for Health and Care Excellence (NICE), 2014; Naughton et al., 2015) was shown to be important for enhancing participants’ engagement with the programme (Davidson et al., 2012; Dixon et al., 2016). The presence of a peer co-facilitator was a key enabler for engaging participants and sourcing experiential knowledge and peer support. The group process of sharing stories and the sense of collective experience is beneficial to families and service users in terms of reducing isolation and increasing knowledge of mental health issues (Petrakis et al., 2014; Poole et al., 2015). However, the study findings do point to how in-group differences (whether family participants are spouses, siblings or parents) may disrupt the communal feeling and mutual identification within the group which is core to engaging participants in shared learning. In these circumstances, providers’ skills in facilitation are critical to cultivate a rapport and communicative space that facilitates group sharing and learning. The kind of knowledge pertinent to spouse, siblings and parents may also differ, therefore, it is also important for facilitators to take this into account during the facilitation process, as this study highlights how, facilitators’ responsiveness to participants’ informational, emotional and practical needs facilitates engagement.

While didactic learning is a useful component of psychoeducation, (particularly in the sessions addressing diagnosis and psychotropic medication), interactions between participants and clinicians proved engaging and beneficial for participants in terms of them being able to access professional knowledge in a more empowered setting. The session on pharmacology was also explicitly highlighted as an important part of the programme in Petrakis et al.’s (2014) study.

While the study findings provide a multi-site, multi-stakeholder perspective and provides useful insights to inform actions to strengthen engagement, they need to be considered in light of the following limitations. In addition to the potential for interpretative, selection and recall bias, the views presented do not reflect what inhibited engagement of those who didn’t attend the programmes. Neither does the sample represent racial and ethnic minorities, and given the evidence that these groups experience more barriers when attempting to engage with services and are more likely to receive poorer quality of care (Alegria et al., 2020; McGuire & Miranda, 2008), this is an area that requires further attention as the EOLAS programmes evolve. As with all qualitative research the transferability of the findings are limited by the organizational context in which the study was conducted.

## Conclusion

Findings from this study illustrate that lack of engagement is not simply due to participant factors but a combination of factors that exist at varying levels of the ecosystem. In addition to highlighting the importance of organisational readiness and leadership, the findings also point to the importance of conducting local needs assessment to inform the design of responsive recruitment and engagement strategies as well as strengthening the capacity of facilitators through sustained training opportunities and accessible mentoring support. While clinicians’ capacity in terms of skill and time was found to be critical in inspiring initial engagement, it was the synergy of peer involvement, facilitator skill, programme content, group process and group composition that was harnessed to sustain engagement with the intervention. Hence, any potential local adaptations to the intervention need to be evaluated.

## Supplementary Information

Below is the link to the electronic supplementary material.

Supplementary file1 (DOC 26 kb)

Supplementary file2 (DOC 64 kb)

Supplementary file3 (DOC 58 kb)

## Data Availability

Unfortunately, we cannot make publicly available our raw data for the following reasons. Given the nature of the study and participants descriptions of their experiences, including experiences of mental distress, making complete transcripts publicly available could compromise confidentiality or participant privacy. In addition, our original ethics permissions did not specify to participants that their data would be made available to third parties.
