# Supplementary material for: Factors Influencing Attendees’ Engagement with Group Psychoeducation: A Multi-stakeholder Perspective
Source: Adm Policy Ment Health. 2022 Jan 6;49(4):539–51. doi: 10.1007/s10488-021-01182-y (PMC9233715; doi:10.1007/s10488-021-01182-y)
Supplement: Supplementary file 1 — Supplementary file1 (DOC 26 kb) [file 10488_2021_1182_MOESM1_ESM.doc]

**Additional File 1: Interview Guide**

General questions that guided all interviews, with minor adaptations depending on the group being interviewed

**Opening questions**

- Some general background information from Interviewee.
- Could you please describe what your role/involvement in EOLAS is/was?

**Challenges**

Can you identify factors which **challenged** you?

- - Intervention characteristics (training, manuals, content, delivery, format, recruitment process)
  - Inner setting (payments, buy-in, leadership, perceived value, relationships, dynamic of co-delivery, support, communication pathways)
  - Provider/facilitator (skill-set, confidence, knowledge, beliefs, adaptability, openness to change, commitment, attitudinal)
  - Service user/family participant (context, access, acceptability, adaptability, attitudinal)

**Strategies to overcome challenges**

- How were/are these challenging factors minimised or resolved?
  - Prompts: Adapt processes, change personnel, change location, secured support

**Facilitators**

Can you describe factors which **facilitated** you?

- - Intervention characteristics (training, content, delivery, format, recruitment process, relative benefit)
  - Inner setting (payments, buy-in, leadership, perceived value, relationships, dynamic of co-delivery, support, communication pathways)
  - Provider/facilitator (skill-set, confidence, knowledge, beliefs, adaptability, openness to change, commitment, attitudinal)
  - Service user/family participant (context, access, acceptability, adaptability, attitudinal)
