# Supplementary material for: Factors Influencing Attendees’ Engagement with Group Psychoeducation: A Multi-stakeholder Perspective
Source: Adm Policy Ment Health. 2022 Jan 6;49(4):539–51. doi: 10.1007/s10488-021-01182-y (PMC9233715; doi:10.1007/s10488-021-01182-y)
Supplement: Supplementary file 2 — Supplementary file2 (DOC 64 kb) [file 10488_2021_1182_MOESM2_ESM.doc]

Additional file 2: Overview of themes, subthemes and illustrative quotes

**Theme 1: Participant**

| **Primary theme** | **Subtheme** | **Illustrative quotes** | |
| --- | --- | --- | --- |
| **Theme 1: Participant** | **Motivation** | **Enabler** | [...] We all had the same reason as wanting to move forward in our lives and wanted to take steps to ground ourselves well and wanted to know more about mental illness in order to develop coping strategies and to liaise with the mental health teams and professionals more beneficially. SP3 (II)  […] once we’d heard about it we were keen to learn as much as possible. We always were asking questions if you like at the hospital (Laughs). […]. The minute I heard about it I decided oh absolutely we have to do that. FP12 (II) |
|  |  | **Barrier** | I suppose they [service users] don’t want to sign up for anything, we find it hard to get them to change, make one little change, you know to their routine or their week. CO16 (II) |
|  | **Readiness** | **Barrier** | […] people need to be in a really good place to do it. That I think is another kind of impeding factor, you have to be really well to do it. Because I’ve seen people struggle with it and I’ve seen people unwell […] But you know everybody has to kind of function at a higher level. […] people who are newly diagnosed - it’s nearly too early, people are only coming to terms with the diagnoses, they might be linked in with the EIP [Early Intervention Psychosis] program. CO16 (II)  Now there was actually one person, it was a couple and the husband struggled with it. And found it very stressful and didn’t come to the very last one. And there is that kind of thing, their daughter was newly diagnosed. And they were in a really difficult space. CF4 (FG) |
|  | **Desire to distance oneself from mental health services** | **Barrier** | I just had no interest in hearing. I was off meds that year and I wanted nothing to do with mental health (services)just because I wanted to kind of convince myself I’m past all that. And I just wasn’t open to listening to it. SP2 (II) |
|  | **Stigma** | **Barrier** | […] it’s such a small town […]it seemed to carry just that little bit more of a stigma, […] I think it’s just everyone knows everyone, that’s what one lady said to me, she says everyone knows everyone. [….] one lady said I was really nervous I’d know people in here. And the others had said that they were the same, because she said ‘I think if I did know someone I wouldn’t say anything’. CO1 (II)  I was late diagnosed, I was 36 so I had my own stigma. You know around that so I wouldn’t have wanted to have been seen to be going to clinics and if I went to outpatient appointments I used to look around. SF14 (FG) |
|  | **Confidentiality** | **Barrier** | I suppose I was worried some of this information could get back [to their relative] and would not be reported as I had said it and I wasn’t sure what the reaction would be. And I was a bit fearful in that respect, so I kept it limited let’s put it that way. FP3 (II) |
|  | **Family Dynamics** | **Barrier** | We have a family member who rang up the day before we were starting and she said that she wanted, she was all signed up to do it. But her daughter wanted to come along [to the family group].. Because she didn’t want her talking about her. She wanted to make sure she wasn’t saying anything about her. So in the end the lady didn’t come herself, you know because obviously the service user can’t come to the family one. SF11 (FG) |

**Theme 2: Programme related factors**

| **Primary theme** | **Subtheme** | **Illustrative quotes** | |
| --- | --- | --- | --- |
| **Theme 2: Programme related factors** | **Training Programme** | **Enabler** | When we did the training, an emphasis was that it’s not about you, or your mental health experience or your story. It’s more about the people [participants], The people in the room. So it’s not an opportunity for you to talk about yourself. So it’s more to get them to talk about themselves. SF10 (FG) |
|  | **Manuals** | **Enabler** | […] if I have a communication problem with my [family member], I go to the section on communication and I look at it and I say, well how should I approach that. That doesn’t say about spending money or, if she was looking for more money from us right. But it told you how to communicate. So I do refer to the manual a lot because you never remember it all. But that’s what the manual is for. FF8 (FG)  […] didactic book learning is very important. But there’s also a wealth of learning in terms of experience. And having a dialogue between how things are supposed to be according to the books and how things really feel, and their experience in real life. And having that kind of a dialogue back and forth, cross referencing back and forth is the most effective way to make the book useful and applicable to my life. SH1 (II) |
|  |  | **Barrier** | I suppose our first few ones that we rolled out there was a huge amount of referrals for people with psychosis and bi-polar disorder, but we find the content is very much more psychosis, kind of unusual beliefs, all that type of thing, so [...] sometimes when you get to the end [of the programme] you would see it in their evaluation that [...] they would just say that they found that there wasn’t enough on their own [bipolar] diagnosis. CO1 (II)  I found flipping through books could be distracting for the person next to them or the book would sometimes take away, it was like as if they needed the books to be able to talk whereas I think it would have been much more free flowing had it been maybe on a powerpoint type thing CF2 (II) |
|  | **Peer Facilitation** | **Enabler** | I think it was really positive to have somebody with a lived experience of it and I think the, the family and friends, it really resonated with them [...] CO6 (II)  And I think that is the reason why EOLAS works, is because of that co facilitation. And that’s who they look towards. It’s not the person, the professional whoever that is. It’s the person who has the son with a diagnosis of schizophrenia. CF4 (FG) |
|  | **Guest Speakers** | **Enabler** | […] to actually have the psychiatric team to come and talk to you on a one to one basis […] and answer your questions, I never thought that would be possible. […]. They [fellow participants] all felt exactly the same way as me. It [attendance of a psychiatrist] appeared to have a big impact on them and they were delighted to be able to ask the questions you know. They were very vocal and they did ask whatever they wanted to know. SF2 (II)  There is something about that consultant going in to the group and meeting people at a more egalitarian level rather than actually sitting, not literally, but metaphorically at least, sitting behind the desk and having a list of symptoms and, and if the families as well only ever meet the consultant in that context of either crisis or bad news or […] for them to meet the consultant in a more social atmosphere and a more, a more level playing field is a really powerful thing, I’ve noticed that several times […] CF7 (FG) |
|  | **Group Context** | **Enabler** | […] being a participant in a lot of ways supported my recovery. Because you feel like you’ve got something to contribute. And you see and hear other people’s frustrations and sharing of their lived experiences. It was very supportive in that regard. Even though EOLAS is not therapy. It’s not that it’s group therapy, or intended to be or anything like that. Just having people chat and interact with each other and you know over the tea break or what have you is therapeutic in and of itself. SF7 (II) |
|  |  | **Barrier** | When I went to the course, there were other family members there and I found people were very hesitant to speak [...] I remember everybody was very cautious so I felt very exposed in a way [...] FP9 (II) |
|  | **Group Make-Up** | **Barrier** | [...] but that to me would be the major drawback that I was the only one with a spouse, they were adult but they were children adults, child adult…but I know, I appreciate that it's quite difficult to set that up [a group for spouses when you are trying to get a group together. FP3 (II) |
|  | **Duration and Timing** | **Barrier** | […] we had it on during the day so for some family members it wasn’t practical to come during the day, which meant I suppose maybe taking time off work or d’you know that kind of stuff. CF2 (II)  […] can we be flexible in it? Is there any way that we can condense this into a shorter period of time because 8 weeks is a huge commitment. Can we run it over 3 half days for people, you know what works well for the person? […] I think there’s a better chance of getting people for shorter periods. […] just even at weekends […] we’re actually talking about on the letter, […] if we had the 3 options over the 8-week period, over 4 weekends whatever way we break it down, and if people don’t actually fill it and send it back to us we know they’re not going to be interested. […] that’s what we’re thinking of going forward. And when we do get some sort of baseline and say ok the majority of people are going for this, let’s just go for it. CO4 (II)  Sure it’s dark, how am I supposed to get there [as no transport]? CF12 (FG): |

**Theme 3: Provider**

| **Primary theme** | **Subtheme** | **Illustrative quotes** | |
| --- | --- | --- | --- |
| **Theme 3: Provider** | **Engaged Clinicians** | **Enabler** | The response to the referral is very important… [Name of coordinator] would actually make contact [with the facilitator] and say you know we’ve got a referral in and just give them [potential attendee] more information and organise and arrange to meet them CF5 (FG) |
|  |  | **Barrier** | But the first meeting...only two people turned up to the first meeting, because as said, they [clinicians] didn’t make the phone calls. The following week they made all the phone calls and we had twelve. And from then on it was grand. FF7 (FG) |
|  | **Facilitator Skillset** | **Enabler** | I suppose it really would be down to the facilitators and their ability to facilitate [...] it is an education group with a process piece…Most of the time it’s just setting up the conversation and the topic and having a process to have the conversation and through hearing what the group is saying the facilitators will have or should have the ability and the knowledge to offer and signpost the required information. So that’s how your group can deal with any subject, because the group is then set up to adapt to the people that are in the group. You know psycho education only works if you are giving and offering the right information at that point in time. CO5 (II) |
|  |  | **Barrier** | Yeah, and I think it’s important that the peer facilitator would be a little bit open to sharing like their, so say the last girl [peer facilitator], was a little bit more boundaried about her own stuff, so we’re gently trying to say if you feel comfortable sharing something, because people get a lot more out of that, so, whereas the other person –he had been on an inpatient unit for seven years and he’s been in community now and there was people like going what, you were in there for seven years, are you serious, you know, and actually with him sharing that kind of stuff it really helped people, and being honest [...] CF10 (FG)  And it is very important that you [peer and clinician] kind of understand each other… you try and support each other. Or that you pick up on each other’s kind of approach and that you kind of try and kind of work in sync [harmony]. I would find it very hard to be with a facilitator who might be selling a different message. Or going kind of.. going rogue, or yea (Laughs). And I’d find that very uncomfortable do you know. CF5 (FG) |

**Theme 4: Organisational**

| **Primary theme** | **Subtheme** | **Illustrative quotes** | |
| --- | --- | --- | --- |
| **Theme 4: Organisational** | **Structural issues** | **Barrier** | I thought it could’ve been better resourced. So in other words it tended to be taking people, adding work onto their daily workload. Instead of saying, right we’ll have a dedicated team to actually roll this out and embed it in the local area, if that makes sense. FF2 (II)  The clinicians are really busy. And they’ve lots of new roles and there’s things happening in the HSE. You know that is affecting you know trying to get these courses run. SF10 (FG) |
|  | **Multiple recovery initiatives** | **Barrier** | It’s [EOLAS] kind of taking a back foot in the last eighteen months, Advancing Recovery in Ireland, recovery principles [education], they’re new and then the exciting recovery college, and we kind of lost the momentum as a result […] CF9 (FG) |
|  | **Lack of advertising/visibility of progrmmes within service** | **Barrier** | [...] it’s not advertised enough. FF3 (FG)  And I don’t think an awful lot of people really know about it, I don’t think there was that much information out there, that it [EOLAS] was on like for family members and service users. FP1 (II) |
